# Determination of the longitudinal sensitivity of the AVVQ-Brazil Quality of Life Questionnaire to non-surgical treatment of chronic venous disease

**DOI:** 10.1590/1677-5449.190048

**Published:** 2019-11-18

**Authors:** Flávia de Jesus Leal, Renata Cardoso Couto, Guilherme Benjamin Brandão Pitta, Solange Andreoni

**Affiliations:** 1 Universidade Federal de São Paulo – UNIFESP, São Paulo, SP, Brasil.; 2 Universidade Estadual de Ciências da Saúde de Alagoas – UNCISAL, Maceió, AL, Brasil.

**Keywords:** sensitivity and specificity, quality of life, venous insufficiency

## Abstract

**Background:**

Sensitivity determines the efficiency and quality of construction of an assessment measure, but studies of the subject are scarce in the literature.

**Objective:**

To evaluate the longitudinal sensitivity of the AVVQ-Brazil to clinical changes after treatment for chronic venous disease (CVD).

**Methods:**

A longitudinal intervention study, with 112 chronic venous patients receiving elective treatment, assessed with CEAP, VAPS, AVVQ-Brazil, and VEINES-QOL/Sym at pre-treatment (baseline) and post-treatment (4 weeks). Differences in the scores for the scales at different times were evaluated using Student’s *t* test for paired samples and Wilcoxon’s *z*, which were also used to asses individuals by CEAP grade and assessment time. Effect size, confidence intervals, and partial η^2^ were used to determine the sensitivity of changes in scale scores over time. Correlations between changes in the scores of the same scales and between different scales were measured using Pearson coefficients, Spearman coefficients, and Kendall’s tau-b coefficient.

**Results:**

The mean age of the patients was 59.51 years. The majority were female (82.1%), with standing for prolonged periods (49.1%), had completed secondary (22.3%) or higher (25%) education, and had CEAP C2 (28.6%) or C6 (32.1%) clinical severity. The following results were observed: mean scale scores reduced from baseline to post-treatment, except for the Extent of Varicosities domain of the AVVQ-Brazil and the VEINES-QOL/Sym scales; sensitivity was low for the AVVQ-Brazil and its domains and for the VAPS, and was from low to high for the other scales; there were improvements or maintenance of CEAP grade after treatment; and moderate to excellent correlations between changes in scale scores over time.

**Conclusions:**

The AVVQ-Brazil is sensitive to longitudinal clinical changes after treatment for CVD and is an important measure for assessment of QoL and of disease severity in Brazil.

## INTRODUCTION

New lifestyle habits, technical and scientific advances in healthcare, and increasing life expectancy have made chronic diseases and their discomforts commonplace.^1^ One of the most frequent, chronic venous disease (CVD), is the cause of considerable burden on health services, caused by its complications that limit daily activities and the ability to work and impose suffering on those affected, deteriorating their quality of life (QoL).^2^^,^ 3

There are few studies in the literature that are designed to validate and evaluate the sensitivity of QoL questionnaires in CVD.^4^ One such questionnaire is the original version of the Aberdeen Varicose Vein Questionnaire (AVVQ).^5^ Its sensitivity was tested by Smith et al.^5^ only in 1999, although developed in 1993 by Garratt et al.^6^, who observed significant improvements in health after surgery for varicose veins, indicating moderate to high sensitivity, and by Lattimer et al.^7^ in 2014, who observed a significant reduction in total score after endovenous treatment of varicose veins.

In addition to assessing QoL in CVD, the AVVQ can also measure disease severity, has demonstrated good validity, consistency, and reproducibility in its home country,^5^^,^ 6 ^,^ 8 and has been used in several different studies. Translated and validated for Dutch, it proved to be reliable and valid for assessment of CVD.^9^ To facilitate adoption, an on-line version was launched in the United Kingdom and was found to be an acceptable measurement instrument, easy to use, reliable, and valid.^10^

After validation for Brazil, the Aberdeen Varicose Veins Questionnaire was released in Brazilian Portuguese (AVVQ-Brazil), with evidence of its validity, internal consistency, and reproducibility for the Brazilian population,^3^^,^ 11 ^,^ 12 with sensitivity to aspects of CVD such as signs and symptoms, and adequacy for assessment of compromised QoL dimensions.^3^ The first stages of validation of the AVVQ-Brazil, comprising translation, cultural adaptation, and evaluation of internal consistency, reproducibility, and validity, have already been conducted and were published in the Jornal Vascular Brasileiro in 2012 and 2015, but it still remains to determine its sensitivity to clinical changes.^3^^,^ 11 ^,^ 12

The effectiveness of assessment questionnaires is established by their psychometric indices, which are determined as part of the validation process,^13^ and good sensitivity is a necessary element of adequacy for use.^14^

Sensitivity, responsiveness, or capacity to respond is the capacity to measure important changes over time in a given concept and is a determinant factor of the quality of construction of an instrument.^15^ It can be measured in two ways: by studying people in whom true improvement is expected and then calculating the effect size (ES), or by using a criterion of true change and investigating the extent to which the measure is capable of distinguishing between individuals who have or have not undergone true change.^16^

Considering the scarcity of studies to determine the sensitivity of CVD assessment questionnaires and its low availability in Brazil, the need to supplement AVVQ-Brazil validation, the importance of QoL assessment to reveal changes in clinical variables during therapeutic interventions and for selection and interpretation of results clinical studies of CVD, this study was conducted to determine the longitudinal sensitivity of the AVVQ-Brazil to clinical changes among patients with CVD undergoing non-surgical treatment.

## METHOD

Questionnaire validation study, approved by the Research Ethics Committee at the Universidade Federal de São Paulo (UNIFESP), São Paulo, SP, Brazil, with approval certificate CAAE nº 401.997. Patients were selected by non-probabilistic sampling from May 2015 to October 2017 at the angiology and vascular surgery clinic of a hospital, a clinic, and an integrated referral center.

In contrast with other psychometric indices, there are no definitive sample size criteria for validation of sensitivity, so the sample size was based on the samples employed for testing sensitivity in other studies.^5^^,^ 7 ^,^ 9 ^,^ 17 ^-^ 19

We recruited patients of both sexes, with CVD confirmed by examination by a vascular surgeon, C2-C6 CEAP clinical classification (Clinical Manifestations, Etiologic factors, Anatomic Distribution of Disease, Pathophysiologic Findings), identified at the first consultation by clinical examination of the lower limbs, considering the limb with the highest grade, and scheduled for elective treatment. Therapeutic management was also defined by the vascular surgeon (sclerotherapy with glucose and/or foam, Unna boot and/or dressings), but patients were not grouped on the basis of the treatment chosen.

Exclusion criteria were age < 18 or ≥ 60 years, cognitive dysfunction according to the mini mental state examination (MMSE); concomitant arterial and lymphatic conditions; diabetes and neuropathies; erysipelas, lymphangitis, acute deep vein thrombosis, chronic obstructive postthrombotic syndrome, ulcers of any non-venous etiology; psychiatric disorders and/or dementia (medical diagnosis). Patients unable to speak or understand Portuguese were also excluded.

People who met the inclusion criteria were invited to take part during the first contact and, if they accepted, were interviewed after signing a free and informed consent form.

Previously trained observers collected responses to questionnaires, which were self-administered or administered by interview, assessing patients at two different times. At baseline, (pre-treatment), they were assessed for CEAP grade, a visual analog pain scale (VAPS) was administered, and they answered the quality of life questionnaires AVVQ-Brazil and the Brazilian versions of the Venous Insufficiency Epidemiological and Economic Study – Quality of Life/Symptoms (VEINES-QOL/Sym) scales. At 4 weeks (post-treatment), patients were reassessed for CEAP, and the VAPS, the AVVQ-Brazil, and the VEINES-QOL/Sym were administered again.

The VEINES-QOL produces two different scores, both of which will have a mean score of 50 for the sample assessed. If the sample is assessed at two different times, the mean score for the whole sample will be identical at both times, meaning the score cannot be used to investigate changes over time.^20^ In view of this, the original scoring calculation was not adopted and an intrinsic scoring method (iVEINES-QOL/Sym) proposed by Bland et al.^21^ was used instead. This system scores all item responses as 1, 2, 3, ..., k, where k is the number of response categories for the item, and then recodes each item score as (i-1)/(k-1), producing a score between 0 and 1, which is averaged over all questions to give a total score and then multiplied by 100 and rounded to the nearest integer, giving a more manageable score.

Distribution of patients according to CEAP was compared between the two assessment times using the Wilcoxon *z* nonparametric test. Differences in scale scores were compared using the Wilcoxon *z* nonparametric test and Student’s parametric paired *t* test. Sensitivity to changes was compared by calculating effect sizes (ES) based on the standard deviation (SD) of the change, on the baseline SD, on the partial η^2^, and on the confidence interval (CI) of the change. Correlations between changes in scale scores were assessed using Pearson, Spearman, and Kendall’s τ-b correlation coefficients, with significance level at p < 0.05.

Sensitivity to clinical changes detects changes in specific situations,^22^ so it is determined by testing predefined hypotheses and calculating the ES.^23^ The longitudinal sensitivity of the AVVQ-Brazil was assessed in terms of the ES for the before and after change in total and domain scores against the VAPS, the VEINES-QOL/Sym, and the CEAP grade (clinical), also analyzing the CI of the change. The following supplementary data were also investigated: age, gender, educational level, therapeutic procedure, and habitual position.

Effect sizes were calculated using the Methods for the Behavioral, Educational, and Social Sciences (MBESS) package in the R statistical program, as described by Kelley,^24^ using the ci.sm command (Confidence Interval for the Standardized Mean) from the manual^24^ and dividing the difference between the means for the scores at the two assessment times by the SD for the difference (ES = difference between means/SD of the difference). Cohen proposes the following reference values for ES: ES ≥ 0.8 - high sensitivity; ES ≥ 0.5 to < 0.8 - moderate sensitivity; and ES ≥ 0.20 to < 0.50 - low sensitivity.^25^ Another method of calculating effect size that is used in clinical studies is to divide the mean of the differences between the two assessment times (end-baseline) by the SD of the variable at baseline.^15^ Both methods were used to calculate ES for changes, 4 weeks after the intervention, for total and domain AVVQ-Brazil scores, VAPS, and the VEINES-QOL/Sym.

Sensitivity was also evaluated by partial η^2^, another different measure of effect size suggested by Cohen,^26^ which is the proportion of total variance explained at the two assessment times. Test power is expressed as a percentage (%), indicating the probability of detecting an effect greater than or equal to that observed with the sample size and significance level employed (5%), assuming the effect to be true. Test power qualifies the sample size of the study for the purpose of detecting the difference found. Approximate values for partial η^2^, according to Cohen,^26^ are: partial η^2^ > 0.13 is a large ES; 0.02 to 0.13 is a moderate ES; and 0.00 to 0.02 is a small ES.

## RESULTS

A sample of 118 patients with CVD was recruited, six of whom were later excluded, five because they did not answer the AVVQ-Brazil at the second assessment time and one because of an MMSE score below the cutoff point.

Mean patient age was 59.51 years (SD = 14.03). The majority were female (82.1%), spent prolonged periods standing up (49.1%), had completed secondary education (26.8%) or higher education (25%), and had CEAP clinical severity of C2 (28.6%) or C6 (32.1%).

The AVVQ-Brazil, VEINES-QOL/Sym, and VAPS were administered to assess QoL, signs and symptoms, and pain, respectively, at baseline and after 4 weeks. There was an overall reduction in mean scores for all scales, except for the Extent of Varicosities domain of the AVVQ-Brazil and the iVEINES-QOL/Sym (Table 1).

**Table 1:** Descriptive summary of the AVVQ-Brazil, VAPS, and VEINES-QOL/Sym scales, by assessment times.

**Scale**	**Assessment**	**n**	**Mean**	**Standard deviation**	**Minimum**	**Maximum**	**Median**
Total AVVQ-Brazil score	Baseline	112	24.57	11.86	1.52	63.40	23.88
	4 weeks	112	20.29	9.45	0.52	45.74	20.00
AVVQ-Brazil, Pain and Dysfunction	Baseline	112	33.80	30.90	0.00	100.00	25.30
	4 weeks	112	18.74	22.17	0.00	100.00	11.05
AVVQ-Brazil, Esthetic Appearance	Baseline	112	51.17	36.17	0.00	100.00	56.76
	4 weeks	112	43.14	39.35	0.00	100.00	35.15
AVVQ-Brazil, Extent of Varicosities	Baseline	112	21.50	15.49	0.93	67.81	18.10
	4 weeks	112	22.78	15.07	0.93	77.11	19.66
AVVQ-Brazil, Complications	Baseline	112	17.28	20.45	0.00	91.42	5.94
	4 weeks	112	12.92	15.99	0.00	66.00	7.02
VAPS	Baseline	112	4.10	3.17	0.00	10.00	4.50
	4 weeks	112	2.73	3.07	0.00	10.00	2.00
iVEINES-QOL	Baseline	112	55.11	22.09	9.80	94.00	54.80
	4 weeks	112	65.75	20.36	10.40	99.20	65.50
iVEINES-Sym	Baseline	112	59.48	24.99	7.00	100.00	60.50
	4 weeks	112	66.85	22.46	10.00	100.00	69.00

n = sample size.

Table 2 shows the results for changes over time, with similar values in the Wilcoxon test and Student’s *t* test, with statistically significant results for the total AVVQ-Brazil (p < 0.001) and its Pain and Dysfunction (p < 0.001), Esthetic Appearance (p = 0.020 and p = 0.017), and Complications (p = 0.015 and p = 0.011) domains, VAPS (p < 0.001), iVEINES-QOL (p < 0.001), and iVEINES-Sym (p < 0.001). However, the result for the Extent of Varicosities domain on the AVVQ-Brazil was not statistically significant (p = 0.363 and p = 0.347).

**Table 2:** Comparative analysis with the Wilcoxon *z* test and Student’s *t* test for paired samples to assess differences over time on the AVVQ-Brazil, VAPS, and iVEINES-QOL/Sym scales.

**Scale**	**n**	**Change median (4 weeks - baseline)**	**Wilcoxon’s *z***	**p**	**Change median (4 weeks - baseline)**	**Standard deviation of the change**	**95%CI of the change**	**t**	**p**	**Observed power (%)** **with alpha = 0.05**
AVVQ-Brazil, total score	112	-2.62	-3.61	<0.001	-4.28	11.44	-6.42 to -2.13	-3.96	< 0.001	97.51
AVVQ-Brazil, Pain and Dysfunction	112	-6.62	-4.73	< 0.001	-15.06	29.16	-20.52 to -9.60	-5.47	< 0.001	99.97
AVVQ-Brazil, Esthetic Appearance	112	0.00	-2.31	0.020	-8.04	35.06	-14.60 to -1.47	-2.43	0.017	67.18
AVVQ-Brazil, Extent of Varicosities	112	0.93	-0.91	0.363	1.28	14.31	-1.40 to 3.96	0.94	0.347	15.48
AVVQ-Brazil, Complications	112	0.00	-2.42	0.015	-4.36	17.90	-7.70 to -1.00	-2.58	0.011	72.32
VAPS	112	0.00	-3.64	< 0.001	-1.38	3.68	-2.06 to -0.69	-3.95	< 0.001	97.47
iVEINES-QOL	112	9.80	-5.93	< 0.001	10.64	16.76	7.50 to -13.78	6.72	< 0.001	99.99
iVEINES-Sym	112	7.00	-3.86	< 0.001	7.37	20.87	3.46 to -11.28	3.74	< 0.001	95.95

n = sample size; p = significance value.

The ES values indicated low sensitivity for the AVVQ-Brazil and its domains and for the VAPS, and low to moderate sensitivity for the iVEINES-QOL/Sym. Partial η^2^ values indicated a large ES for the Pain and Dysfunction domain of the AVVQ-Brazil (0.212) and the iVEINES-QOL (0.289); moderate for VAPS (0.123), for the total AVVQ- Brazil scores (0.124) and its Esthetic Appearance (0.050) and Complications (0.056) domains, and for the iVEINES-Sym (0.112); but small for the Extent of Varicosities domain (0.008), indicating, in general, low to high sensitivity (Table 3).

**Table 3:** Effect sizes after 4 weeks for the AVVQ-Brazil, VAPS, and iVEINES-QOL/SYM scales.

**Scale**	**Change mean (4 weeks - baseline)**	**Standard deviation of the change**	**Standard deviation baseline**	**Effect size (SD of the change)**	**95%CI** **Effect size (SD of the change)** ^*^	**Effect size** (Baseline SD)*	**partial η^2^**
AVVQ-Brazil, Total score	-4.28	11.44	11.86	-0.374	-0.565 to -0.181	-0.361	0.124
AVVQ-Brazil, Pain and Dysfunction	-15.06	29.16	30.90	-0.517	-0.713 to -0.318	-0.487	0.212
AVVQ-Brazil, Esthetic Appearance	-8.04	35.06	36.17	-0.229	-0.416 to -0.041	-0.222	0.050
AVVQ-Brazil, Extent of Varicosities	1.28	14.31	15.49	0.089	-0.097 to 0.275	0.083	0.008
AVVQ-Brazil, Complications	-4.36	17.90	20.45	-0.243	-0.431 to -0.055	-0.213	0.056
VAPS	-1.38	3.68	3.17	-0.373	-0.564 to -0.181	-0.435	0.123
iVEINES-QOL	10.64	16.76	22.09	0.635	0.431 to -0.837	0.482	0.289
iVEINES-Sym	7.37	20.87	24.99	0.353	0.161 to -0.543	0.295	0.112

* SD = standard deviation used.

As shown in Table 4, there were significant changes in CEAP grades at 4 weeks post-treatment (p < 0.001). It was observed that 33.9% (95%CI [25.7%-43.0%], n = 38) of the patients had improved (their CEAP grade had reduced); 62.5% (95%CI [53.3%-71.1%], n = 70) were at the same CEAP grade; and 3.6% (95%CI [1.2%-8.3%], n = 4) had worsened. Therefore, a majority of the patients maintained or reduced their CEAP.

**Table 4:** Distribution of patients by CEAP grade at the two assessment times.

**CEAP**		**4 weeks**							**Wilcoxon signed rank test for paired samples**	
**baseline**		**1**	**2**	**3**	**4**	**5**	**6**	**Total**	***z***	**p**
2	n	9	21	2	0	0	0	32	-5.15	< 0.001
	% of overall total	8.0	18.8	1.8	0.0	0.0	0.0	28.6		
3	n	1	5	8	1	0	0	15		
	% of overall total	0.9	4.5	7.1	0.9	0.0	0.0	13.4		
4	n	0	3	4	17	0	0	24		
	% of overall total	0.0	2.7	3.6	15.2	0.0	0.0	21.4		
5	n	0	0	0	0	4	1	5		
	% of overall total	0.0	0.0	0.0	0.0	3.6	0.9	4.5		
6	n	0	0	0	0	16	20	36		
	% of overall total	0.0	0.0	0.0	0.0	14.3	17.9	32.1		
Total	n	10	29	14	18	20	21	112		
	% of overall total	8.9	25.9	12.5	16.1	17.9	18.8	100		

n = sample size; *z* = test statistic; p = significance value.

It can be observed in Table 5 that the Pearson, Spearman, and Kendall’s τ-b correlation coefficients were similar, with statistically significant correlations between changes and scores on the following scales: Total AVVQ-Brazil score and VAPS (p < 0.001), Total AVVQ-Brazil score and CEAP (p = 0.003), Pain and Dysfunction and VAPS (p < 0.05), Pain and Dysfunction and CEAP (p = 0.002), Esthetic Appearance and VAPS (p < 0.05), Esthetic Appearance and iVEINES-QOL (p < 0.05), Esthetic Appearance and CEAP (p < 0.05), Extent of Varicosities and VAPS (p < 0.05) and total AVVQ-Brazil score and all of its domains (p < 0.001), indicating, in general, correlations in the range of moderate to excellent. None of the other correlations exhibited statistically significant values (p > 0.05).

**Table 5:** Correlations between changes in total AVVQ-Brazil score and its domain scores with each other and with VAPS, iVEINES-QOL, and iVEINES-Sym.

			Pearson		Spearman		Kendall’s τ-b	
Change in	Change in	n	Correlation	p	Correlation	p	Correlation	p
Total AVVQ-Brazil score	VAPS	112	0.498	< 0.001	0.475	< 0.001	0.358	< 0.001
Total AVVQ-Brazil score	iVEINES-QOL	112	0.065	0.652	0.173	0.225	0.113	0.267
Total AVVQ-Brazil score	iVEINES-Sym	112	-0.290	0.039	-0.232	0.102	-0.177	0.098
Total AVVQ- Brazil score	CEAP	112	0.427	0.002	0.416	0.002	0.345	0.003
AVVQ-Brazil, Pain and Dysfunction	VAPS	112	0.363	0.009	0.342	0.014	0.260	0.011
AVVQ-Brazil, Pain and Dysfunction	iVEINES-QOL	112	-0.075	0.603	0.115	0.421	0.082	0.421
AVVQ-Brazil, Pain and Dysfunction	iVEINES-Sym	112	-0.309	0.028	-0.201	0.157	-0.154	0.151
AVVQ-Brazil, Pain and Dysfunction	CEAP	112	0.425	0.002	0.445	0.001	0.370	0.002
AVVQ-Brazil, Esthetic Appearance	VAPS	112	0.384	0.005	0.372	0.007	0.274	0.007
AVVQ-Brazil, Esthetic Appearance	iVEINES-QOL	112	0.254	0.072	0.389	0.005	0.278	0.006
AVVQ-Brazil, Esthetic Appearance	iVEINES-Sym	112	-0.153	0.283	-0.082	0.569	-0.064	0.550
AVVQ-Brazil, Esthetic Appearance	CEAP	112	0.375	0.007	0.350	0.012	0.290	0.013
AVVQ-Brazil, Extent of Varicosities	VAPS	112	0.392	0.004	0.438	0.001	0.322	0.002
AVVQ-Brazil, Extent of Varicosities	iVEINES-QOL	112	-0.050	0.726	-0.053	0.711	-0.037	0.718
AVVQ-Brazil, Extent of Varicosities	iVEINES-Sym	112	-0.109	0.447	-0.049	0.731	-0.029	0.788
AVVQ-Brazil, Extent of Varicosities	CEAP	112	0.166	0.245	0.163	0.254	0.135	0.250
AVVQ-Brazil, Complications	VAPS	112	0.166	0.245	0.131	0.360	0.094	0.360
AVVQ-Brazil, Complications	iVEINES-QOL	112	0.009	0.950	-0.073	0.612	-0.046	0.651
AVVQ-Brazil, Complications	iVEINES-Sym	112	-0.135	0.344	-0.147	0.302	-0.119	0.268
AVVQ-Brazil, Complications	CEAP	112	0.149	0.297	0.137	0.337	0.114	0.332
VAPS	iVEINES-QOL	112	0.171	0.229	0.216	0.127	0.167	0.117
VAPS	iVEINES-Sym	112	-0.121	0.399	-0.034	0.812	-0.030	0.792
VAPS	CEAP	112	0.203	0.153	0.209	0.142	0.181	0.140
iVEINES-QOL	iVEINES-Sym	112	-0.168	0.238	-0.190	0.182	-0.148	0.184
iVEINES-QOL	CEAP	112	0.136	0.343	0.386	0.005	0.332	0.006
iVEINES-Sym	CEAP	112	-0.180	0.207	-0.153	0.284	-0.139	0.280
Total AVVQ-Brazil score	AVVQ-Brazil, Pain and Dysfunction	112	0.781	<0.001	0.762	<0.001	0.585	< 0.001
Total AVVQ-Brazil score	AVVQ-Brazil, Esthetic Appearance	112	0.740	<0.001	0.731	<0.001	0.563	< 0.001
Total AVVQ-Brazil score	AVVQ-Brazil, Extent of Varicosities	112	0.603	<0.001	0.564	<0.001	0.414	< 0.001
Total AVVQ-Brazil score	AVVQ-Brazil, Complications	112	0.589	<0.001	0.520	<0.001	0.369	< 0.001
AVVQ-Brazil, Pain and Dysfunction	AVVQ-Brazil, Esthetic Appearance	112	0.548	<0.001	0.483	<0.001	0.345	< 0.001
AVVQ-Brazil, Pain and Dysfunction	AVVQ-Brazil, Extent of Varicosities	112	0.230	0.104	0.232	0.102	0.152	0.120
AVVQ-Brazil, Pain and Dysfunction	AVVQ-Brazil, Complications	112	0.219	0.123	0.137	0.339	0.116	0.235
AVVQ-Brazil, Esthetic Appearance	AVVQ-Brazil, Extent of Varicosities	112	0.130	0.365	0.168	0.239	0.109	0.265
AVVQ-Brazil, Esthetic Appearance	AVVQ-Brazil, Complications	112	0.215	0.130	0.221	0.119	0.157	0.109
AVVQ-Brazil, Extent of Varicosities	AVVQ-Brazil, Complications	112	0.486	<0.001	0.423	0.002	0.300	0.002

n = sample size; p = significance value.

## DISCUSSION

Questionnaires for assessment of the impact on QoL of CVD or its treatments should be tested prospectively, in order to investigate patient experience by means of psychometric analyses of sensitivity.^27^

A systematic review by Aber et al.^27^ included studies undertaken from 1993 to 2016, analyzing the psychometric properties of CVD questionnaires, identified disparate degrees of psychometric rigor, concluding that only the original AVVQ assessed important psychometric domains in detail. This underscores the need to determine the sensitivity of the AVVQ-Brazil, since sensitivity is an important factor in validation that enables appropriate use of an instrument, but one which has not yet been tested for the AVVQ-Brazil. Another point highlighted in that review was the variation in post-treatment follow-up period in the studies analyzed, ranging from immediately after intervention to 12 months afterwards.^27^

In another review, it was observed that when the original AVVQ was administered at 3 weeks and at 3 months, scores worsened during the first few weeks after treatment, before improving for 4-6 weeks. It was therefore concluded that 3 weeks was too early to observe improvement in the less responsive items on the questionnaire.^7^

A study employing the original AVVQ to assess the results of treatment after 1, 12, 24, and 36 weeks reported a mild deterioration in AVVQ score 1 week post-treatment, improving significantly by 12 months.^28^ As such, a very short assessment time may not reveal significant post-treatment improvements, because of the immediate effects of the therapeutic procedure itself, including pain, and it is therefore necessary to allow more time to detect improvements.

In view of the lack of standardization in the literature with regard to methodology and follow-up time for determination of sensitivity, for this study it was decided that patients would be reassessed at 4 weeks post-treatment.

### Post-treatment changes

Clinical guidelines recommend using QoL to evaluate the results of treatment for varicose veins and to facilitate patient monitoring.^29^^,^ 30 Data show that these treatments significantly improve the health of patients, when scores on the original AVVQ are compared before and after surgery, and also show that those whose scores were lower before treatment (less severe) benefit less from the intervention.^31^

Compared with surgery, sclerotherapy and thermal ablation treatments are associated with earlier return to work, lower duration of incapacity, and less pain.^32^ Those findings are consistent with the results of this study, in which, irrespective of the treatment chosen, there was an overall reduction in both total AVVQ-Brazil scores and its domain scores, and also in VAPS scores, with the exception of the Extent of Varicosities domain and the VEINES-QOL/Sym scales, indicating improvement in QoL and pain over time.

The increase in Extent of Varicosities domain score may be because this is an item that is difficult to change, involving patient perception, and, in cases where not all of the varicose veins were treated, then perceptions may not have changed. Furthermore, this is a domain that is not so sensitive to post-treatment response as other domains, and it is possible that some patients had limited ability to answer the questions it contains. These limitations would be related to item 1 (diagram), in which some patients (especially older patients) may have difficulty drawing their varicose veins and little body awareness, and item 5 (use of elastic stockings), because of low compliance caused by the difficulty involved in putting them on, the discomfort caused, and the high cost. According to Castro-Ferreira et al.,^33^ esthetic perception is a subjective characteristic, that is difficult to measure.

With regard to the increase in the VEINES-QOL/Sym scores, this probably occurred because these questionnaires prioritize the general aspect of CVD, capturing other aspects less,^34^ in contrast with the AVVQ-Brazil, which reflects disease severity in terms of symptoms and clinical signs.^3^ Additionally, the majority of the patients in this study had higher CEAP grades (4, 5, or 6), which means that they have very often been living with the disease for a long time and have undergone palliative treatments without definitive resolution, influencing their general and psychological condition.^34^

It is recommended that patients be classified by CEAP grade to guide therapeutic decisions, but this classification is not very sensitive to slight changes in disease severity.^35^ Notwithstanding, when we analyzed CEAP clinical severity at the two assessment times, we observed changes in the categories of some patients, primarily in the direction of improvement (reduction). However, in more severe CVD, small to moderate changes in QoL may remain undetected, leading to variability in the results, which increases significantly as disease severity increases and is responsible for discrepancies observed in the relationship between QoL and CEAP grade.^36^

### Sensitivity to changes over time

Smith et al.,^5^ conducted a validation study of the original AVVQ measuring QoL and the effect of surgery on QoL in venous patients assessed 6 weeks after surgery and assessed the sensitivity of the questionnaire using standardized response methods, reporting a value of 0.55, indicative of moderate sensitivity. In contrast, this study found low sensitivity for total AVVQ-Brazil score and its domain scores (ES ≥ 0.20 to < 0.50) and also for the VAPS (ES < 0.20), and moderate for VEINES-QOL/Sym (ES = 0.635 and ES = 0.353, respectively), calculated according to the ES of the change. The partial η^2^ values also showed ES varying from small to large for the scales studied. The small ES may have been caused by the great variability of the sample.

It is clear that there is minimal psychometric evidence on CVD questionnaires,^27^ and few studies have tested the sensitivity of the original AVVQ to clinical change, particularly using ES calculations.

It has been recommended that statistical significance should be presented together with ES and CIs, because the p values that result from statistical tests do not provide information on the magnitude of the difference detected. It is therefore necessary to report ES, which gives the statistical tests meaning, emphasizes their power, reduces the risk that mere sample variation will be interpreted as a true relationship, increases reporting of non-significant results, and aggregates the knowledge of several different studies; preferably presented in relation to the average, for greater precision in sample-based estimates.^20^ In this study, we have reported ES values and their CIs for changes over time in the scales investigated, for determination of sensitivity.

Effect size is not affected by sample size, but the precision of its 95%CI is, so that, generally, the greater the sample, the larger the precision.^20^ Therefore, this study, by reporting ES and 95%CI, will provide useful knowledge with relation to the ideal sample size for further studies, since prior knowledge of these ESs can be used to calculate statistical power and to estimate the appropriate sample size.^37^

Since there is no consensus on values for the magnitude of ES, they should not be rigidly categorized and interpreted, and it is important to consider the area of investigation and the context of variables in real life, obtain ES from intervention studies and compare the effects observed with those previously established in the area.^20^^,^ 37 It is known that the greater the ES, the greater the impact on the central variable of the study and the greater the importance of its contribution to the issue under analysis.^37^

To help with this interpretation of results, Cohen suggested cutoff points for ES. However, these values can vary depending on the area of study and should only be used when there is no better basis for estimating a classification of ES for the dataset being studied. Other authors argue that ES should be interpreted depending on the benefits that can be reaped at a given cost, and should not be classified numerically. Thus, if a given intervention is of low cost, but high benefit, a smaller effect size can have great practical significance or, in contrast, may not have such a great significance, so it is the researcher’s responsibility to analyze the adequacy of results.^37^

Since no preexisting classification of ES established in the same area as that investigated in this study was found, Cohen’s estimates were used to interpret and analyze the effect size values. However, observing the cost-benefit relationship, in which the therapeutic procedures employed are of low cost and result in benefits in terms of improved QoL and CVD symptoms, as demonstrated in the literature and observed in this study using scales, it is clear that, despite the small ES, the effect can be considered of practical significance.

Finally, calculating ES can be useful to compare effects, in a single study, between variables measured on different scales, or for metanalysis.^37^

### Correlations between changes on the scales

Some CVD patients are asymptomatic, while many have symptoms such as feelings of heaviness, pain, edema and itching, with a negative effect on QoL.^38^ In the present study, weak to moderate and statistically significant correlations were observed between changes in total AVVQ-Brazil score and also Pain and Dysfunction and Esthetic Appearance domain scores and changes in VAPS scores and CEAP grades, which occurred concomitantly and indicated that lower specific quality of life (higher values on the AVVQ-Brazil) was associated with higher values on the pain scale and higher CEAP grades, and that treatment can modify these elements. There was also a weak to moderate statistically significant correlation between changes in the Extent of Varicosities domain score and changes in VAPS score, demonstrating change in patients’ perceptions of their varicose veins concomitant with changes in the level of pain.

We did not find any studies correlating changes in specific QoL, measured with the AVVQ, with changes in CEAP clinical severity during the post-therapeutic period. Although the CEAP classification descriptively analyzes the severity of CVD at a single point in time, it is not sensitive to changes in severity over time or post-treatment. Despite this, the changes seen in this study in those whose CEAP class increased may have occurred because some patients may or may not rapidly progress to a more severe level of the disease and develop post-treatment recurrence with the sequential progress of the disease. Some of the patients remaining at the same CEAP grade may have been because certain clinical grades are resistant to change (C4) or permanently static (C5). There is not yet a clear explanation for this in the literature.^36^

This study is subject to the limitations of only assessing changes in QoL at 4 weeks after treatment and of only studying a single group of patients. Thus, longer follow-up periods cannot be analyzed and there was no control group in which subjects did not undergo intervention. Future studies should deal with these issues.

## CONCLUSIONS

The AVVQ-Brazil is sensitive to clinical changes occurring 4 weeks after treatment for CVD.
