# Supplementary material for: Determination of the longitudinal sensitivity of the AVVQ-Brazil Quality of Life Questionnaire to non-surgical treatment of chronic venous disease
Source: J Vasc Bras. 2019 Nov 18;18:e20190048. doi: 10.1590/1677-5449.190048 (PMC6880614; doi:10.1590/1677-5449.190048)
Supplement: Translation

# Translation: Verificação da sensibilidade longitudinal do questionário de qualidade de vida AVVQ-Brasil ao tratamento não cirúrgico da doença venosa crônica

**Authors:** Flávia de Jesus Leal, Renata Cardoso Couto, Guilherme Benjamin Brandão Pitta, Solange Andreoni

**Affiliations:** 1 Universidade Federal de São Paulo – UNIFESP, São Paulo, SP, Brasil.; 2 Universidade Estadual de Ciências da Saúde de Alagoas – UNCISAL, Maceió, AL, Brasil.

**Keywords:** sensibilidade e especificidade, qualidade de vida, insuficiência venosa

## Abstract

**Contexto:**

A sensibilidade determina a eficiência e a qualidade de construção de uma medida de avaliação, mas estudos sobre o tema são escassos na literatura.

**Objetivos:**

Verificar a sensibilidade longitudinal do AVVQ-Brasil às mudanças clínicas após tratamento da doença venosa crônica (DVC).

**Métodos:**

Estudo de intervenção longitudinal, 112 doentes venosos crônicos em tratamento eletivo, avaliados por CEAP, EVA dor, AVVQ-Brasil e VEINES-QOL/Sym, pré (basal) e pós-tratamento (4 semanas). As diferenças nas pontuações das escalas entre momentos foram avaliadas pelos testes *t* Student pareado e *z* de Wilcoxon, que avaliou também indivíduos por CEAP e momentos de avaliação. Tamanho do efeito, intervalo de confiança e η^2^ parcial verificaram a sensibilidade às mudanças nas pontuações das escalas ao longo do tempo. A correlação entre mudanças nas pontuações de mesmas escalas e entre diferentes escalas foi mensurada pelos coeficientes de Pearson, de Spearman e tau_b de Kendall.

**Resultados:**

A idade média dos pacientes era de 59,51 anos. A maioria era do sexo feminino (82,1%), com postura em ortostatismo prolongado (49,1%), tinha ensino médio (22,3%) ou superior completo (25%), e apresentava gravidade clínica CEAP C2 (28,6%) ou C6 (32,1%). Foram observados os seguintes resultados: diminuição das médias das escalas entre momentos, exceto no domínio Extensão da Varicosidade do AVVQ-Brasil e no VEINES-QOL/Sym; sensibilidade pequena para AVVQ-Brasil e seus domínios e para EVA dor, e de pequena a grande para as demais escalas; melhora ou manutenção do CEAP pós-tratamento; e correlações moderadas a excelentes entre mudanças nas escalas ao longo do tempo.

**Conclusões:**

O AVVQ-Brasil é longitudinalmente sensível às mudanças clínicas pós-tratamento da DVC, sendo medida importante de avaliação da QV e da gravidade da doença no Brasil.

## INTRODUÇÃO

Novos hábitos de vida, avanços técnico-científicos da saúde e aumento na expectativa de vida tornaram mais habituais as doenças crônicas e seus desconfortos^1^. Uma das mais frequentes, a doença venosa crônica (DVC), gera grande demanda nos serviços de saúde, devido a suas complicações, resultando em limitação de atividades diárias e laborativas e sofrimento aos acometidos e influenciando na qualidade de vida (QV)^2^^,^ 3.

Na literatura existem poucos estudos desenvolvidos para validar e avaliar a sensibilidade de questionários de QV na DVC^4^, tais como o questionário Aberdeen Varicose Vein Questionnaire (AVVQ) original. Teve sua sensibilidade avaliada por Smith et al. 5 somente em 1999, embora desenvolvido em 1993 por Garratt et al.^6^, que observaram melhora significativa na saúde pós-cirurgia de varizes, indicando moderada a alta sensibilidade, e por Lattimer et al.^7^ em 2014, que encontraram uma significativa redução na pontuação total após tratamento endovenoso das varizes.

Além de avaliar QV na DVC, o AVVQ mede a gravidade da doença, apresentando validade, consistência e reprodutibilidade boas no país de origem^5^^,^ 6 ^,^ 8, e é utilizado em vários estudos. Traduzido e validado para a língua holandesa, confirmou ser confiável e válido na avaliação da DVC^9^. Para facilitar sua adoção, surgiu uma versão *on-line* no Reino Unido, a qual demonstrou ser um instrumento de medição aceitável, fácil de utilizar, confiável e válido^10^.

Na validação para nosso país, surgiu o Questionário Aberdeen para Veias Varicosas no Brasil (AVVQ-Brasil), o qual se evidenciou válido, consistente internamente e reprodutível para a população brasileira^3^^,^ 11 ^,^ 12, e sensível a aspectos da DVC como sinais e sintomas, sendo adequado para a avaliação das dimensões comprometidas da QV^3^. A primeira etapa da validação do AVVQ-Brasil, composta pela tradução, adaptação cultural e determinação da consistência interna, reprodutibilidade e validade, já foi realizada e publicada no Jornal Vascular Brasileiro nos anos de 2012 e 2015, cabendo agora verificar sua sensibilidade às mudanças clínicas^3^^,^ 11 ^,^ 12.

Determina-se a eficiência dos questionários avaliativos por seus índices psicométricos, estabelecidos no processo de validação^13^, sendo que para seu uso adequado é necessária boa sensibilidade^14^.

A análise de sensibilidade, responsividade ou capacidade de resposta consiste em mensurar mudanças importantes ocorridas ao longo do tempo em um conceito, sendo fator determinante de qualidade de construção^15^. Pode ser medida de duas formas: estudando indivíduos com melhora verdadeira esperada calculando o tamanho do efeito (TE), ou usando critérios de mudança verdadeira a fim de investigar o quanto a medida é capaz de distinguir indivíduos que tiveram ou não melhora real^16^.

Considerando a escassez de estudos de sensibilidade de questionários avaliativos da DVC e sua pequena disponibilidade no Brasil, a necessidade de complementação da validação do AVVQ-Brasil, a importância da avaliação da QV para refletir alterações nas variáveis clínicas durante intervenção terapêutica e para a seleção e interpretação de resultados em estudos clínicos sobre DVC, este estudo busca verificar a sensibilidade longitudinal do AVVQ-Brasil às mudanças clínicas de indivíduos com DVC submetidos a tratamento não cirúrgico.

## MÉTODO

Estudo de validação de questionário, aprovado pelo Comitê de Ética em Pesquisa da Universidade Federal de São Paulo (UNIFESP), São Paulo, SP, Brasil, sob CAAE nº 401.997, com pacientes selecionados por amostragem não probabilística entre maio de 2015 e outubro de 2017 em ambulatório de angiologia e cirurgia vascular de um hospital, uma clínica e um centro de referência integrado.

Diferentemente dos demais índices psicométricos, para sensibilidade inexistem critérios definidos nas diretrizes de validação quanto ao tamanho da amostra, sendo este determinado considerando amostras utilizadas para testar sensibilidade em outros estudos^5^^,^ 7 ^,^ 9 ^,^ 17 ^-^ 19.

Foram incluídos pacientes de ambos os sexos com DVC comprovada por avaliação de cirurgião vascular, com base na classificação clínica CEAP (Clinical Manifestations, Etiologic factors, Anatomic Distribution of Disease, Pathophysiologic Findings) C2-C6, identificada na primeira consulta pelo exame clínico dos membros inferiores, considerando o membro de maior classe, e submetidos a tratamento eletivo. A conduta terapêutica também foi definida pelo cirurgião vascular (escleroterapia com glicose e/ou espuma, Bota de Unna e/ou curativos), mas os pacientes não foram agrupados segundo a terapêutica escolhida.

Foram excluídos indivíduos com idade < 18 e ≥ 60 anos com alterações cognitivas, avaliadas por meio do miniexame do estado mental (MEEM); alterações arteriais e linfáticas associadas; diabetes e neuropatias; erisipela, linfangite, trombose venosa profunda aguda, síndrome pós-trombótica crônica obstrutiva e úlcera de qualquer etiologia não venosa; distúrbios psiquiátricos e/ou quadro demencial (diagnóstico médico). Além disso, foram excluídos indivíduos sem fala ou compreensão da língua portuguesa.

Os indivíduos que atendiam aos critérios de inclusão foram convidados a participar durante primeiro contato e, em caso de aceite, foram entrevistados, após assinarem termo de consentimento livre e esclarecido.

Observadores previamente treinados coletaram as respostas dos questionários por autoaplicação ou entrevista, avaliando os pacientes em dois momentos. No momento basal (pré-tratamento), foram investigados quanto ao CEAP e quanto à dor pela escala visual analógica para dor (EVA dor), respondendo aos questionários de qualidade de vida AVVQ-Brasil e a versão brasileira do Venous Insufficiency Epidemiological and Economic Study – Quality of Life/Symptom (VEINES-QOL/Sym). Após 4 semanas (pós-tratamento), os pacientes foram reavaliados quanto ao CEAP e EVA dor, respondendo novamente ao AVVQ-Brasil e ao VEINES-QOL/Sym.

O VEINES-QOL origina duas pontuações distintas, cada uma com pontuação média de 50 para a amostra avaliada. Caso a amostra seja avaliada em dois momentos diferentes, a pontuação média geral será idêntica, dificultando a investigação de mudanças ao longo do tempo^20^. Logo, não foi utilizado o cálculo original, optando-se pelo método de pontuação intrínseca (iVEINES-QOL/Sym), proposto por Bland et al.^21^, no qual as questões são pontuadas 1, 2, 3, ..., k, sendo k o número de categorias, e cada pontuação do item i é recodificada para (i-1)/(k-1), possibilitando a cada item pontuação entre 0 e 1 e média sobre as questões para pontuação final, multiplicada por 100, arredondando para número inteiro mais próximo, obtendo-se uma pontuação mais gerenciável.

A distribuição dos pacientes segundo CEAP entre os dois momentos de avaliação foi comparada por meio do teste não paramétrico *z* de Wilcoxon. As diferenças nas pontuações das escalas foram comparadas por meio do teste não paramétrico *z* de Wilcoxon e do teste paramétrico *t* de Student pareado. A sensibilidade às mudanças foi comparada calculando-se os tamanhos de efeito (TEs) com base no desvio padrão (DP) da mudança, no DP basal e no η^2^ parcial e o intervalo de confiança (IC) da mudança. As correlações entre as mudanças das escalas foram avaliadas calculando-se os coeficientes de correlação de Pearson, Spearman e τ_b de Kendall, com nível de significância p < 0,05.

Sensibilidade às mudanças clínicas detecta alterações em situações específicas^22^; avalia-se testando hipóteses pré-definidas e calculando-se o TE^23^. Avaliou-se a sensibilidade longitudinal do AVVQ-Brasil pelo TE da mudança na pontuação total e por domínios, na EVA dor, no VEINES-QOL/Sym e no CEAP (clínica), entre os momentos, analisando também o IC da mudança. Foram investigados os seguintes dados complementares: idade, gênero, escolaridade, procedimento terapêutico e posição assumida.

O TE foi calculado pelo pacote Methods for the Behavioral, Educational, and Social Sciences (MBESS) do programa estatístico R, descrito por Kelley^24^, utilizando comando ci.sm *(*Confidence Interval for the Standardized Mean) do manual^24^ , dividindo-se a diferença entre as médias dos escores nos dois momentos pelo DP dessa diferença (TE = diferença entre as médias/DP da diferença). Segundo Cohen, o TE tem os seguintes valores de referência: TE ≥ 0,8 indica sensibilidade grande; TE ≥ 0,5 a < 0,8, sensibilidade moderada; e TE ≥ 0,20 a < 0,50, sensibilidade pequena^25^. Outro tamanho do efeito utilizado em estudos clínicos é calculado dividindo-se a média das diferenças entre os dois momentos de avaliação (final-basal) pelo DP da variável no momento basal^15^. Calculou-se o TE das mudanças pelos dois métodos, 4 semanas pós-intervenção, para as pontuações do AVVQ-Brasil e de seus domínios, da EVA dor e do VEINES-QOL/Sym.

A sensibilidade foi avaliada também pelo η^2^ parcial, uma medida diferente de tamanho do efeito sugerida por Cohen^26^, que significa a proporção da variância total explicada pelos momentos de avaliação. O poder do teste é expresso em porcentagem (%), indicando a probabilidade de encontrar efeito maior ou igual ao observado com o tamanho da amostra ao nível de significância utilizado (5%), supondo esse efeito verdadeiro. O poder de teste qualifica o tamanho amostral do estudo para detectar a diferença encontrada. Os valores aproximados do η^2^ parcial, segundo Cohen^26^, são os seguintes: η^2^ parcial > 0,13 indica TE grande; entre 0,02 e 0,13, TE moderado; e, entre 0,00 e 0,02, TE pequeno.

## RESULTADOS

Foi selecionada uma amostra de 118 pacientes com DVC, sendo que seis foram posteriormente excluídos, cincos deles por não completarem o AVVQ-Brasil na segunda aplicação, e um deles por não atingido o ponto de corte do MEEM.

A idade média dos pacientes foi de 59,51 anos (DP = 14,03). Além disso, a maioria era do gênero feminino (82,1%), encontrava-se em ortostatismo prolongado (49,1%), tinha ensino médio completo (26,8%) ou superior completo (25%), e apresentava gravidade clínica CEAP C2 (28,6%) e C6 (32,1%).

A aplicação do AVVQ-Brasil, VEINES-QOL/Sym e EVA dor avaliou QV, sinais e sintomas, e dor, respectivamente, a partir das respostas basais e em 4 semanas. Observou-se uma diminuição geral das médias entre os momentos para todas as escalas, exceto para o domínio Extensão da Varicosidade do AVVQ-Brasil e para o iVEINES-QOL/Sym (Tabela 1).

**Tabela 1:** Resumo descritivo das escalas AVVQ-Brasil, EVA dor e VEINES-QOL/Sym, segundo os momentos de avaliação.

**Escala**	**Momento**	**n**	**Média**	**Desvio padrão**	**Mínimo**	**Máximo**	**Mediana**
AVVQ-Brasil total	Basal	112	24,57	11,86	1,52	63,40	23,88
	4 semanas	112	20,29	9,45	0,52	45,74	20,00
AVVQ-Brasil Dor e Disfunção	Basal	112	33,80	30,90	0,00	100,00	25,30
	4 semanas	112	18,74	22,17	0,00	100,00	11,05
AVVQ-Brasil Aparência Estética	Basal	112	51,17	36,17	0,00	100,00	56,76
	4 semanas	112	43,14	39,35	0,00	100,00	35,15
AVVQ-Brasil Extensão da Varicosidade	Basal	112	21,50	15,49	0,93	67,81	18,10
	4 semanas	112	22,78	15,07	0,93	77,11	19,66
AVVQ-Brasil Complicações	Basal	112	17,28	20,45	0,00	91,42	5,94
	4 semanas	112	12,92	15,99	0,00	66,00	7,02
EVA dor	Basal	112	4,10	3,17	0,00	10,00	4,50
	4 semanas	112	2,73	3,07	0,00	10,00	2,00
iVEINES-QOL	Basal	112	55,11	22,09	9,80	94,00	54,80
	4 semanas	112	65,75	20,36	10,40	99,20	65,50
iVEINES-Sym	Basal	112	59,48	24,99	7,00	100,00	60,50
	4 semanas	112	66,85	22,46	10,00	100,00	69,00

n = tamanho da amostra.

Na Tabela 2, que apresenta as mudanças ocorridas ao longo do tempo, verificou-se valores semelhantes nos testes Wilcoxon e *t* de Student, observando-se significância estatística para AVVQ-Brasil total (p < 0,001), domínios Dor e Disfunção (p < 0,001), Aparência Estética (p = 0,020 e p = 0,017) e Complicações (p = 0,015 e p = 0,011), EVA dor (p < 0,001), iVEINES-QOL (p < 0,001) e iVEINES-Sym (p < 0,001). Por outro lado, o domínio Extensão da Varicosidade do AVVQ-Brasil não apresentou significância estatística (p = 0,363 e p = 0,347).

**Tabela 2:** Análise comparativa entre os testes *z* Wilcoxon e *t* de Student pareado para avaliar diferenças no tempo entre as escalas AVVQ-Brasil, EVA dor e iVEINES-QOL/Sym.

**Escala**	**n**	**Mediana da mudança (4 sem - basal)**	***z* de Wilcoxon**	**p**	**Média da mudança (4 sem - basal)**	**Desvio padrão da mudança**	**IC95% da mudança**	**t**	**p**	**Poder observado (%)** **com alfa = 0,05**
AVVQ-Brasil total	112	-2,62	-3,61	<0,001	-4,28	11,44	-6,42 a -2,13	-3,96	< 0,001	97,51
AVVQ-Brasil Dor e Disfunção	112	-6,62	-4,73	< 0,001	-15,06	29,16	-20,52 a -9,60	-5,47	< 0,001	99,97
AVVQ-Brasil Aparência Estética	112	0,00	-2,31	0,020	-8,04	35,06	-14,60 a -1,47	-2,43	0,017	67,18
AVVQ-Brasil Extensão da Varicosidade	112	0,93	-0,91	0,363	1,28	14,31	-1,40 a 3,96	0,94	0,347	15,48
AVVQ-Brasil Complicações	112	0,00	-2,42	0,015	-4,36	17,90	-7,70 a -1,00	-2,58	0,011	72,32
EVA dor	112	0,00	-3,64	< 0,001	-1,38	3,68	-2,06 a -0,69	-3,95	< 0,001	97,47
iVEINES-QOL	112	9,80	-5,93	< 0,001	10,64	16,76	7,50-13,78	6,72	< 0,001	99,99
iVEINES-Sym	112	7,00	-3,86	< 0,001	7,37	20,87	3,46-11,28	3,74	< 0,001	95,95

n = tamanho da amostra; p = valor de significância.

Valores de TE indicam sensibilidade pequena para AVVQ-Brasil e seus domínios e para EVA dor, e sensibilidade de pequena a moderada para o iVEINES-QOL/Sym. Já valores do η^2^ parcial evidenciaram TE grande no domínio Dor e Disfunção do AVVQ-Brasil (0,212) e iVEINES-QOL (0,289); moderado na EVA dor (0,123), no AVVQ-Brasil total (0,124), nos domínios Aparência Estética (0,050) e Complicações (0,056), e no iVEINES-Sym (0,112); e pequeno no domínio Extensão da Varicosidade (0,008), indicando, em geral, sensibilidade pequena a grande (Tabela 3).

**Tabela 3:** Tamanhos de efeito após 4 semanas para as escalas AVVQ-Brasil, EVA dor e iVEINES-QOL/SYM.

**Escala**	**Média da mudança (4 sem - basal)**	**Desvio padrão da mudança**	**Desvio padrão basal**	**Tamanho do efeito (DP da mudança)**	**IC95%** **Tamanho do efeito (DP da mudança)** ^*^	**Tamanho do efeito** (DP basal)*	**η^2^ parcial**
AVVQ-Brasil Total	-4,28	11,44	11,86	-0,374	-0,565 a -0,181	-0,361	0,124
AVVQ-Brasil Dor e Disfunção	-15,06	29,16	30,90	-0,517	-0,713 a -0,318	-0,487	0,212
AVVQ-Brasil Aparência Estética	-8,04	35,06	36,17	-0,229	-0,416 a -0,041	-0,222	0,050
AVVQ-Brasil Extensão da Varicosidade	1,28	14,31	15,49	0,089	-0,097 a 0,275	0,083	0,008
AVVQ-Brasil Complicações	-4,36	17,90	20,45	-0,243	-0,431 a -0,055	-0,213	0,056
EVA Dor	-1,38	3,68	3,17	-0,373	-0,564 a -0,181	-0,435	0,123
iVEINES-QOL	10,64	16,76	22,09	0,635	0,431-0,837	0,482	0,289
iVEINES-Sym	7,37	20,87	24,99	0,353	0,161-0,543	0,295	0,112

* DP = desvio padrão utilizado.

Conforme Tabela 4, ocorreram mudanças significativas no CEAP 4 semanas pós-tratamento (p < 0,001). Observou-se que 33,9% (IC95% [25,7%-43,0%], n = 38) dos pacientes melhoraram, isto é, sua categoria CEAP diminuiu; 62,5% (IC95% [53,3%-71,1%], n = 70) permaneceram com o mesmo CEAP; e 3,6% (IC95% [1,2%-8,3%], n = 4) pioraram. Assim, a maior parte dos pacientes manteve ou diminuiu o CEAP.

**Tabela 4:** Distribuição dos pacientes segundo CEAP nos dois momentos de avaliação.

**CEAP**		**4 semanas**							**Teste de sinal de Wilcoxon pareado**	
**basal**		**1**	**2**	**3**	**4**	**5**	**6**	**Total**	***z***	**P**
2	n	9	21	2	0	0	0	32	-5,15	< 0,001
	% do total geral	8,0	18,8	1,8	0,0	0,0	0,0	28,6		
3	n	1	5	8	1	0	0	15		
	% do total geral	0,9	4,5	7,1	0,9	0,0	0,0	13,4		
4	n	0	3	4	17	0	0	24		
	% do total geral	0,0	2,7	3,6	15,2	0,0	0,0	21,4		
5	n	0	0	0	0	4	1	5		
	% do total geral	0,0	0,0	0,0	0,0	3,6	0,9	4,5		
6	n	0	0	0	0	16	20	36		
	% do total geral	0,0	0,0	0,0	0,0	14,3	17,9	32,1		
Total	n	10	29	14	18	20	21	112		
	% do total geral	8,9	25,9	12,5	16,1	17,9	18,8	100		

n = tamanho da amostra; *z* = estatística do teste; p = valor de significância.

Observa-se, na Tabela 5, coeficientes semelhantes de correlação de Pearson, Spearman e τ_b Kendall, apresentando significância estatística entre as mudanças na pontuação das seguintes escalas: AVVQ-Brasil total e EVA dor (p < 0,001), AVVQ-Brasil total e CEAP (p = 0,003), Dor e Disfunção e EVA dor (p < 0,05), Dor e Disfunção e CEAP (p = 0,002), Aparência Estética e EVA dor (p < 0,05), Aparência Estética e iVEINES-QOL (p < 0,05), Aparência Estética e CEAP (p < 0,05), Extensão da Varicosidade e EVA dor (p < 0,05) e AVVQ-Brasil total e todos os seus domínios (p < 0,001), indicando em geral, correlações de moderadas a excelentes. As demais correlações não apresentaram valores estatisticamente significativos (p > 0,05).

**Tabela 5:** Correlações das mudanças no AVVQ-Brasil total e em seus domínios entre si e com EVA dor, iVEINES-QOL e iVEINES-Sym.

			Pearson		Spearman		τ_b de Kendall	
Mudança em	Mudança em	n	Correlação	p	Correlação	p	Correlação	p
AVVQ-Brasil Total	EVA dor	112	0,498	< 0,001	0,475	< 0,001	0,358	< 0,001
AVVQ-Brasil Total	iVEINES-QOL	112	0,065	0,652	0,173	0,225	0,113	0,267
AVVQ-Brasil Total	iVEINES-Sym	112	-0,290	0,039	-0,232	0,102	-0,177	0,098
AVVQ- Brasil Total	CEAP	112	0,427	0,002	0,416	0,002	0,345	0,003
AVVQ-Brasil Dor e Disfunção	EVA dor	112	0,363	0,009	0,342	0,014	0,260	0,011
AVVQ-Brasil Dor e Disfunção	iVEINES-QOL	112	-0,075	0,603	0,115	0,421	0,082	0,421
AVVQ-Brasil Dor e Disfunção	iVEINES-Sym	112	-0,309	0,028	-0,201	0,157	-0,154	0,151
AVVQ-Brasil Dor e Disfunção	CEAP	112	0,425	0,002	0,445	0,001	0,370	0,002
AVVQ-Brasil Aparência Estética	EVA dor	112	0,384	0,005	0,372	0,007	0,274	0,007
AVVQ-Brasil Aparência Estética	iVEINES-QOL	112	0,254	0,072	0,389	0,005	0,278	0,006
AVVQ-Brasil Aparência Estética	iVEINES-Sym	112	-0,153	0,283	-0,082	0,569	-0,064	0,550
AVVQ-Brasil Aparência Estética	CEAP	112	0,375	0,007	0,350	0,012	0,290	0,013
AVVQ-Brasil Extensão da Varicosidade	EVA dor	112	0,392	0,004	0,438	0,001	0,322	0,002
AVVQ-Brasil Extensão da Varicosidade	iVEINES-QOL	112	-0,050	0,726	-0,053	0,711	-0,037	0,718
AVVQ-Brasil Extensão da Varicosidade	iVEINES-Sym	112	-0,109	0,447	-0,049	0,731	-0,029	0,788
AVVQ-Brasil Extensão da Varicosidade	CEAP	112	0,166	0,245	0,163	0,254	0,135	0,250
AVVQ-Brasil Complicações	EVA dor	112	0,166	0,245	0,131	0,360	0,094	0,360
AVVQ-Brasil Complicações	iVEINES-QOL	112	0,009	0,950	-0,073	0,612	-0,046	0,651
AVVQ-Brasil Complicações	iVEINES-Sym	112	-0,135	0,344	-0,147	0,302	-0,119	0,268
AVVQ-Brasil Complicações	CEAP	112	0,149	0,297	0,137	0,337	0,114	0,332
EVA dor	iVEINES-QOL	112	0,171	0,229	0,216	0,127	0,167	0,117
EVA dor	iVEINES-Sym	112	-0,121	0,399	-0,034	0,812	-0,030	0,792
EVA dor	CEAP	112	0,203	0,153	0,209	0,142	0,181	0,140
iVEINES-QOL	iVEINES-Sym	112	-0,168	0,238	-0,190	0,182	-0,148	0,184
iVEINES-QOL	CEAP	112	0,136	0,343	0,386	0,005	0,332	0,006
iVEINES-Sym	CEAP	112	-0,180	0,207	-0,153	0,284	-0,139	0,280
AVVQ-Brasil total	AVVQ-Brasil Dor e Disfunção	112	0,781	<0,001	0,762	<0,001	0,585	< 0,001
AVVQ-Brasil total	AVVQ-Brasil Aparência Estética	112	0,740	<0,001	0,731	<0,001	0,563	< 0,001
AVVQ-Brasil total	AVVQ-Brasil Extensão da Varicosidade	112	0,603	<0,001	0,564	<0,001	0,414	< 0,001
AVVQ-Brasil total	AVVQ-Brasil Complicações	112	0,589	<0,001	0,520	<0,001	0,369	< 0,001
AVVQ-Brasil Dor e Disfunção	AVVQ-Brasil Aparência Estética	112	0,548	<0,001	0,483	<0,001	0,345	< 0,001
AVVQ-Brasil Dor e Disfunção	AVVQ-Brasil Extensão da Varicosidade	112	0,230	0,104	0,232	0,102	0,152	0,120
AVVQ-Brasil Dor e Disfunção	AVVQ-Brasil Complicações	112	0,219	0,123	0,137	0,339	0,116	0,235
AVVQ-Brasil Aparência Estética	AVVQ-Brasil Extensão da Varicosidade	112	0,130	0,365	0,168	0,239	0,109	0,265
AVVQ-Brasil Aparência Estética	AVVQ-Brasil Complicações	112	0,215	0,130	0,221	0,119	0,157	0,109
AVVQ-Brasil Extensão da Varicosidade	AVVQ-Brasil Complicações	112	0,486	<0,001	0,423	0,002	0,300	0,002

n = tamanho da amostra; p = valor de significância.

## DISCUSSÃO

Questionários de avaliação do impacto da DVC ou seus tratamentos na QV devem ser testados prospectivamente, a fim de investigar a experiência do paciente a partir de análises psicométricas de sensibilidade^27^.

Revisão sistemática de Aber et al.^27^ envolvendo estudos realizados entre 1993 e 2016 que analisaram as propriedades psicométricas de questionários para DVC verificou rigor distinto na avaliação psicométrica, concluindo que o AVVQ original era o único instrumento que avaliava em detalhes domínios psicométricos importantes. Isso fortalece a necessidade de verificação da sensibilidade do AVVQ-Brasil, por tratar-se de fator importante para a sua validação e que permite o uso adequado do instrumento, mas que ainda não testado para o AVVQ-Brasil. Outro ponto a se destacar na revisão acima mencionada é que o tempo de acompanhamento pós-terapêutica variou entre os estudos analisados, sendo que o menor tempo foi imediatamente após a intervenção e o maior foi 12 meses após^27^.

Em outra revisão, observou-se que os escores do AVVQ original, quando avaliados em 3 semanas e 3 meses, pioravam nas primeiras semanas pós-terapêutica, antes de melhorar por 4-6 semanas. Dessa forma, percebeu-se que 3 semanas era muito cedo para serem observadas melhorias em perguntas menos responsivas do questionário^7^.

Estudo utilizando o AVVQ original para avaliar resultados de tratamento nos tempos 1, 12, 24 e 36 semanas relatou ligeira deterioração da pontuação do AVVQ 1 semana pós-tratamento, melhorando significativamente aos 12 meses^28^. Assim, um tempo muito pequeno de avaliação pode não representar mudança significativa pós-tratamento, devido aos efeitos imediatos do próprio procedimento terapêutico, como dor, havendo portanto necessidade de maior tempo para detecção da melhoria.

Diante da falta padronização na literatura sobre metodologia e tempo de acompanhamento para verificação da sensibilidade, neste estudo decidiu-se que os pacientes seriam reavaliados 4 semanas pós-tratamento.

### Mudanças pós-tratamento

Diretrizes clínicas recomendam usar QV para avaliar os resultados do tratamento de varizes e auxiliar no acompanhamento dos pacientes^29^^,^ 30. Dados mostram que esse tratamento melhora significativamente a saúde dos pacientes, quando comparadas pontuações do AVVQ original pré e pós-cirurgia, e que aqueles com pontuações mais baixas pré-tratamento (menos graves) eram menos beneficiados com a intervenção^31^.

Comparadas à cirurgia, escleroterapia e terapias de ablação térmica associam-se com retorno laborativo mais rápido, menor duração da incapacidade e menos dor^32^. Tais achados estão em consonância com os deste estudo, no qual, independente da escolha terapêutica, houve em geral diminuição na pontuação total do AVVQ-Brasil e dos seus domínios e na EVA dor, exceto no domínio Extensão da Varicosidade e no VEINES-QOL/Sym, indicando melhoria da QV e da dor ao longo do tempo.

O aumento na pontuação do domínio Extensão da Varicosidade pode ter ocorrido por este ser um item difícil de modificar, envolvendo percepção do paciente, e, nos casos em que não tenham sido tratadas todas as varizes, sua percepção pode não ter se modificado. Além disso, trata-se de um domínio não tão sensível à resposta pós-tratamento como os demais domínios, e pode haver possíveis limitações dos pacientes em responder suas questões. Tais limitações estão relacionadas à questão 1 (diagrama), na qual alguns pacientes (especialmente o de mais idade) podem apresentar dificuldade para desenhar suas varizes e pouca percepção corporal, e à questão 5 (uso de meias elásticas), devido à baixa adesão decorrente da dificuldade de vesti-las, do desconforto provocado e do alto custo. Segundo Castro-Ferreira et al.^33^, percepção estética é uma característica subjetiva, sendo portanto, dificilmente mensurável.

Quanto ao aumento na pontuação do VEINES-QOL/Sym, o fato provavelmente aconteceu porque esse questionário prioriza o aspecto geral da DVC, capturando menos outros aspectos^34^, em oposição ao AVVQ-Brasil, que reflete a gravidade da doença pelos sintomas e sinais clínicos^3^. Além disso, a maioria dos pacientes deste estudo apresenta CEAP mais elevado (4, 5, 6), o que significa que eles muitas vezes lidam com a doença há muito tempo e foram submetidos a tratamentos paliativos sem resolução definitiva, influenciando o seu perfil geral e psicológico^34^.

Recomenda-se categorização dos pacientes pelo CEAP para nortear decisões terapêuticas; porém, essa categorização é pouco sensível a alterações leves na gravidade da doença^35^. Contudo, ao analisarmos a gravidade clínica CEAP nos dois momentos deste estudo, observamos mudança na categoria de alguns pacientes, principalmente no sentido da melhora (redução). Entretanto, na DVC mais grave, mudanças pequenas a moderadas na QV podem permanecer ocultas, levando à variabilidade de resultado, que cresce significativamente com o aumento da gravidade da doença e é responsável por discrepâncias observadas relação entre mudanças na QV e CEAP^36^.

### Sensibilidade às mudanças ao longo do tempo

Smith et al.^5^, em estudo validativo do AVVQ original para determinar a QV e o efeito da cirurgia sobre esta em pacientes venosos acompanhados 6 semanas pós-cirurgia, avaliaram a sensibilidade do questionário por meios padronizados de resposta, encontrando valor 0,55, indicativo de sensibilidade moderada. Em discordância, este estudo encontrou sensibilidade pequena para pontuação total do AVVQ-Brasil e de seus domínios (TE ≥ 0,20 a < 0,50) e EVA dor (TE < 0,20), moderada para VEINES-QOL/Sym (TE = 0,635 e TE = 0,353, respectivamente), calculada pelo TE da mudança. Também, pelos valores η^2^ parcial, foi observado TE variando entre pequeno a grande nas escalas estudadas, podendo esse TE pequeno ter ocorrido devido à grande variabilidade da amostra.

Está claro haver mínimas evidências psicométricas sobre os questionários para DVC^27^, sendo encontradas poucas pesquisas que testassem a sensibilidade do AVVQ original às mudanças clínicas, em especial pelo cálculo do TE.

Há recomendações de associar a significância estatística à apresentação do TE e dos ICs, pois valores de p, resultados dos testes estatísticos, não informam sobre a magnitude de uma diferença. Deve-se então, reportar o TE, que dá significado aos testes estatísticos, enfatiza o poder deles, reduz o risco de mera variação amostral interpretada como relação real, aumenta o relato de resultados não significativos e acumula conhecimento de vários estudos, devendo ser apresentado em relação à média para maior precisão de estimativas que se baseiam em amostras^20^. Neste estudo, apresentaram-se valores de TE e seus ICs referentes às mudanças ocorridas ao longo do tempo nas escalas avaliadas, para verificação da sensibilidade.

O TE não é afetado pelo tamanho da amostra, mas a precisão do seu IC95% sim, de forma que, habitualmente, quanto maior a amostra, maior a precisão^20^. Dessa forma, este estudo apresentando TE e IC95% proporcionará conhecimento do tamanho da amostra ideal para a realização de novos estudos, pois a observação prévia dos TEs pode servir de base para o cálculo do poder estatístico e a estimativa do tamanho amostral adequado^37^.

Sem consenso sobre valores da magnitude do TE, sua categorização e interpretação não devem ser rígidas, sendo importante considerar áreas de investigação e contexto de variáveis da realidade, obter o TE a partir de estudos de intervenção e comparar os efeitos encontrados com aqueles previamente estabelecidos na área^20^^,^ 37. Sabe-se que, quanto maior o TE, maior o impacto causado pela variável central do estudo e mais importante sua contribuição para a questão analisada^37^.

Ajudando nessa interpretação dos resultados, Cohen sugeriu pontos de corte para TE. Contudo, esses valores podem variar de acordo com a área de estudo e somente devem ser usados quando não for encontrada base melhor para estimar a classificação do TE para o conjunto de dados trabalhados. Outro autor defende que o TE deve ser dependente dos benefícios a serem alcançados a determinado custo, não devendo ser classificado numericamente. Assim, havendo custos baixos em determinada intervenção, porém com benefícios altos, um valor menor de efeito pode ter muita significância prática e, contrariamente, também pode não ter essa significância, cabendo ao pesquisador analisar a adequação dos resultados^37^.

Não encontrando classificação do TE previamente estabelecida dentro da área investigada neste estudo, utilizou-se a estimativa de Cohen para interpretar e analisar os valores encontrados. Entretanto, observando a relação custo-benefício, em que os procedimentos terapêuticos utilizados apresentam baixo custo e trazem benefícios na melhora da QV e dos sintomas da DVC, já comprovados na literatura e observados por meio das escalas do estudo, percebe-se que, apesar do TE pequeno, o efeito pode ser considerado de significância prática.

Por fim, o cálculo do TE pode ser útil para comparar efeitos, em um único estudo, entre variáveis medidas em escalas diferentes ou em metanálises^37^.

### Correlações entre as mudanças das escalas

Na DVC, alguns pacientes são assintomáticos, enquanto muitos apresentam sintomas como sensação de peso, dor, edema e prurido, afetando negativamente a QV^38^. No presente estudo, observaram-se correlações de fracas a moderadas e estatisticamente significativas entre mudanças na pontuação total do AVVQ-Brasil e de seus domínios Dor e Disfunção e Aparência Estética e mudanças na EVA dor e no CEAP, as quais ocorreram concomitantemente e indicaram menor qualidade de vida específica (valor mais alto no AVVQ-Brasil) associada a maiores valores da dor e CEAP, sendo que o tratamento pode modificar esses aspectos. Também houve correlação fraca a moderada estatisticamente significativa entre mudança na pontuação do domínio Extensão da Varicosidade e mudança na EVA dor, demonstrando modificação na percepção dos pacientes quanto às suas varizes concomitante com mudança nos níveis de dor.

Não encontramos estudos correlacionando mudanças na QV específica, medida por meio do AVVQ, com mudanças da gravidade clínica CEAP no período pós-terapêutico. A classificação CEAP, embora analise descritivamente a gravidade da DVC em um único ponto no tempo, é insensível à mudança na gravidade ao longo do tempo ou no pós-tratamento. Apesar disso, neste estudo essa mudança pode ter ocorrido naqueles que aumentaram a classe CEAP, porque alguns pacientes podem ou não progredir rapidamente para um nível mais grave da doença e desenvolver recorrência pós-tratamento com progressão sequencial da doença. A permanência de alguns dos pacientes no mesmo CEAP pode ter ocorrido pois alguns graus clínicos são resistentes a mudanças (C4) ou permanentemente estáticos (C5). A explicação disso ainda não está clara na literatura^36^.

O estudo teve como limitações o fato de avaliar mudanças na QV apenas 4 semanas pós-terapêutica e estudar um único grupo de pacientes. Assim, não foram analisados tempos maiores de acompanhamento e não houve um grupo controle, no qual os indivíduos não fossem submetidos à intervenção. Estudos futuros devem atentar para tais questões.

## CONCLUSÃO

O AVVQ-Brasil é sensível às mudanças clínicas ocorridas 4 semanas pós-tratamento da DVC.
